# Primary Intrathoracic Ectopic Papillary Thyroid Carcinoma, Presenting With Thoracic Spine Metastasis: A Case Presentation and Literature Review

**DOI:** 10.7759/cureus.55329

**Published:** 2024-03-01

**Authors:** Rodhan Khthir, Nash B Binegar

**Affiliations:** 1 Endocrinology, Diabetes and Metabolism, University of North Dakota School of Medicine and Health Sciences, Bismarck, USA; 2 Endocrinology, Diabetes and Metabolism, Sanford Health, Bismarck, USA

**Keywords:** mediastinal primary thyroid cancer, advanced thyroid cancer, sqstm1-ntrk3 fusion, metastatic papillary thyroid cancer, ectopic thyroid tissue

## Abstract

Thyroid cancer in ectopic thyroid tissue is a very rare entity. We report a patient with papillary thyroid cancer arising from upper mediastinal ectopic thyroid tissue. The patient presented with thoracic spine metastasis with cord compression.

The patient was a 67-year-old woman, who presented with upper back pain. Magnetic resonance imaging (MRI) showed suspected metastatic disease in the second and third thoracic vertebrae (T2 and T3). She underwent laminectomy and decompression surgery at the T1-T3 level. The final pathology report showed metastatic thyroid carcinoma with papillary features. She underwent external beam radiation to the affected spine. Computerized tomography (CT) scan of the chest, abdomen, and pelvis showed a 3.0 × 2.8 × 2.3 cm soft-tissue mass in the left superior mediastinum extending into the supraclavicular region. Fluorodeoxyglucose-positron emission tomography (FDG-PET) scan showed hypermetabolic foci in the upper mediastinum. Fine needle aspiration (FNA) of the upper mediastinal mass was consistent with papillary thyroid cancer. Molecular testing from the FNA sample using *Thyroseq V3* showed *SQSTM1NTRK3* chromosomal rearrangement. A total thyroidectomy was performed. Pathology of the resected thyroid was benign. Pathology of the mediastinal mass showed a papillary thyroid carcinoma with focal tall cell features, forming a 4 × 2.5 × 2.5 cm mass. Surgery was followed by ablation with 100 millicuries (mci) of radioactive iodine (I-131) and external beam radiation.

This case highlights the presentation of primary intrathoracic papillary thyroid cancer with SQSTM1-NTRK3 chromosomal rearrangement and the challenges in the diagnosis and management of this unique case. This patient had a very aggressive disease presentation that required multimodal treatment, including thoracic spine decompression, total thyroidectomy, primary intrathoracic goiter resection, high-dose radioactive iodine treatment, and external beam radiation to the affected spine area. SQSTM1-NTRK3 chromosomal rearrangement can be targeted by medications such as larotrectinib and endtrectinib.

## Introduction

This article was previously presented as a meeting abstract at the 2022 Endocrine Society meeting on June 12, 2022.

The thyroid gland originates from the fusion of the median and lateral thyroid anlage [1]. The median thyroid anlage begins to develop around the second week of gestation from the second pharyngeal arch. As the tissue descends toward the heart, a bilobed diverticulum forms with the thyroglossal duct forming between the lobes [1]. The thyroglossal duct connects the tissue to the tongue. As the thyroid continues to descend, the thyroglossal duct disappears. Around this same time, the lateral thyroid anlage fuses with the median around the fifth week of development [1]. As the thyroid continues to descend, it stops immediately anteriorly to the trachea by the seventh week of development [1]. Ectopic thyroid tissue can be encountered anywhere from the base of the skull to the lower neck [1]. It is rarely seen in the mediastinum. The term intrathoracic goiter can have varying definitions and has evolved. Intrathoracic goiters are usually believed to be thyroid tissue present in the mediastinum [2]. Secondary intrathoracic goiters are extensions of thyroid tissue below the normal position of the thyroid gland with maintained connections and a shared blood supply. A primary intrathoracic goiter shares no connection with the thyroid gland and is typically supplied by intrathoracic vessels. These primary intrathoracic goiters are usually present in the anterior superior mediastinum and might be normal or goitrous thyroid tissue [2]. Primary intrathoracic goiters account for less than 1% of all intrathoracic goiters [2]. Treatment for both primary and secondary intrathoracic goiters has been debated among active surveillance, radioiodine ablation, and surgical intervention. The preferred treatment for symptomatic primary intrathoracic goiters is surgical resection [3]. Potential symptoms of intrathoracic goiters include neck masses, dyspnea, and dysphagia [4]. Among the reported cases of primary intrathoracic goiters, cases of malignant intrathoracic goiters have been scarce.

We report a patient with papillary thyroid cancer originating from the primary intrathoracic goiter of the upper mediastinum. The patient presented with thoracic spine metastasis and spinal cord compression. Molecular testing showed SQSTM1-NTRK3 chromosomal rearrangement. There was no evidence of malignant nodules in the thyroid gland when the patient underwent total thyroidectomy.

## Case presentation

The patient is a 67-year-old woman who presented at another healthcare facility with upper back pain for a few weeks. The pain was progressive and moderate in severity with no history of trauma and no history of fever or night sweats. She reported no weakness or sensory changes. Initial laboratory tests showed no leukocytosis, hypercalcemia, or renal impairment. The thyroid function test was normal. Magnetic resonance imaging (MRI) showed suspected metastatic disease in the second and third thoracic vertebrae (T2 and T3). There was an epidural extension of the tumor with cord compression and severe stenosis at the level of T2. She underwent laminectomy and decompression surgery from T1 to T3. The final pathology report showed metastatic thyroid carcinoma with papillary features. She underwent external beam radiation to the affected vertebrae to control the pain.

Thyroid ultrasound showed an oval-shaped hypoechoic lesion within the left thyroid lobe measuring 1.2 × 0.9 × 0.8 cm. The lesion was well circumscribed without echogenic foci. A computerized tomography (CT) scan of the chest showed a 3.0 × 2.8 × 2.3 cm soft-tissue mass in the left superior mediastinum extending into the supraclavicular region. The lesion was separate from the left lobe of the thyroid. CT of the abdomen and pelvis and MRI of the brain showed no evidence of metastatic disease.

**Figure 1:**
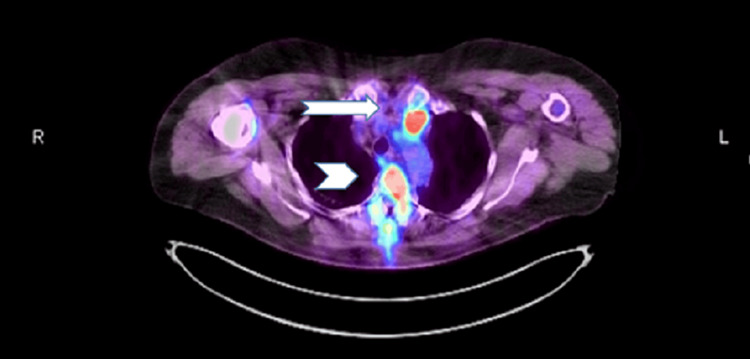
Left upper mediastinal mass (arrow) and vertebral metastasis (arrowhead) with high FDG uptake. FDG: fluorodeoxyglucose

**Figure 2:**
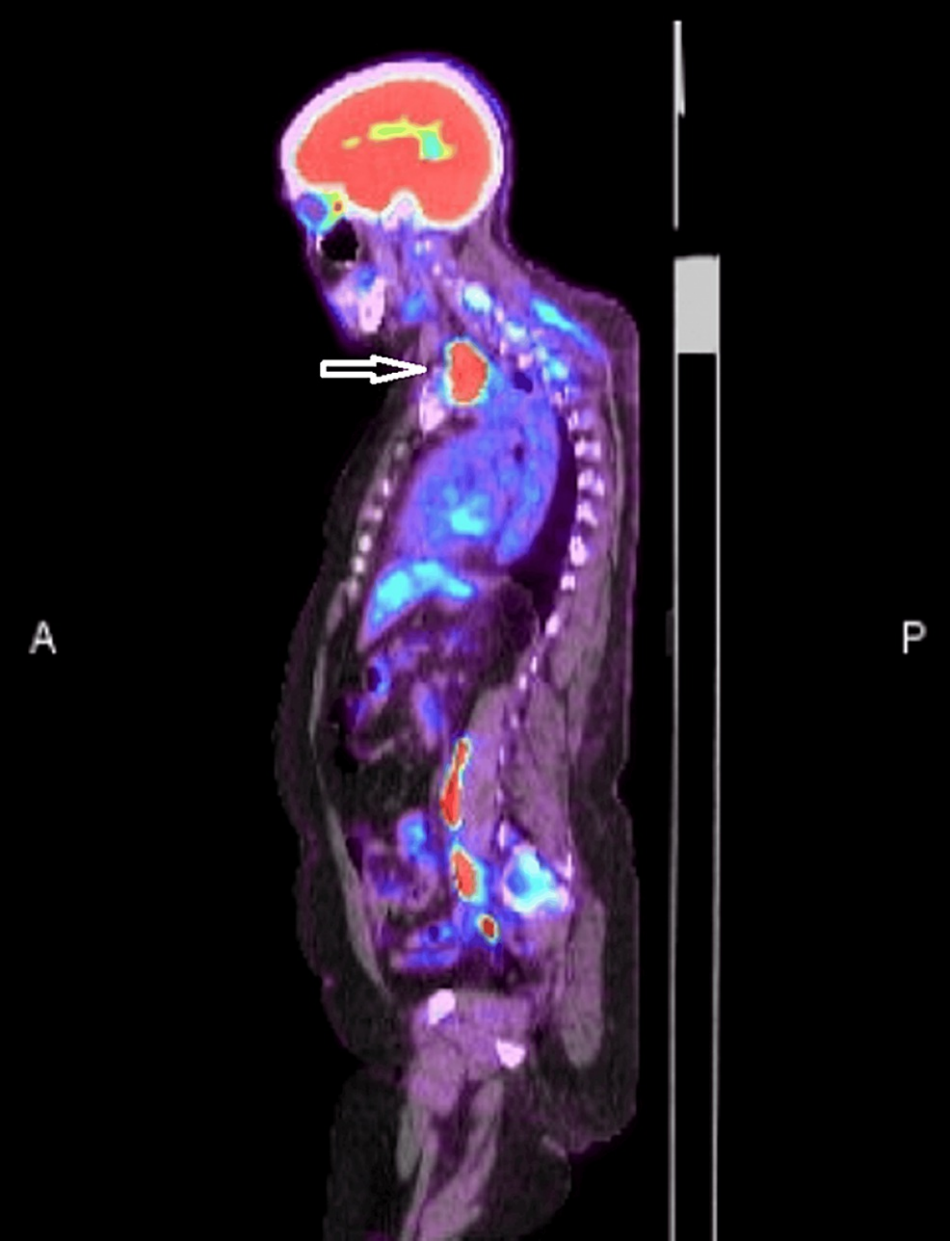
Upper mediastinal mass with high FDG uptake. FDG: fluorodeoxyglucose

**Figure 3:**
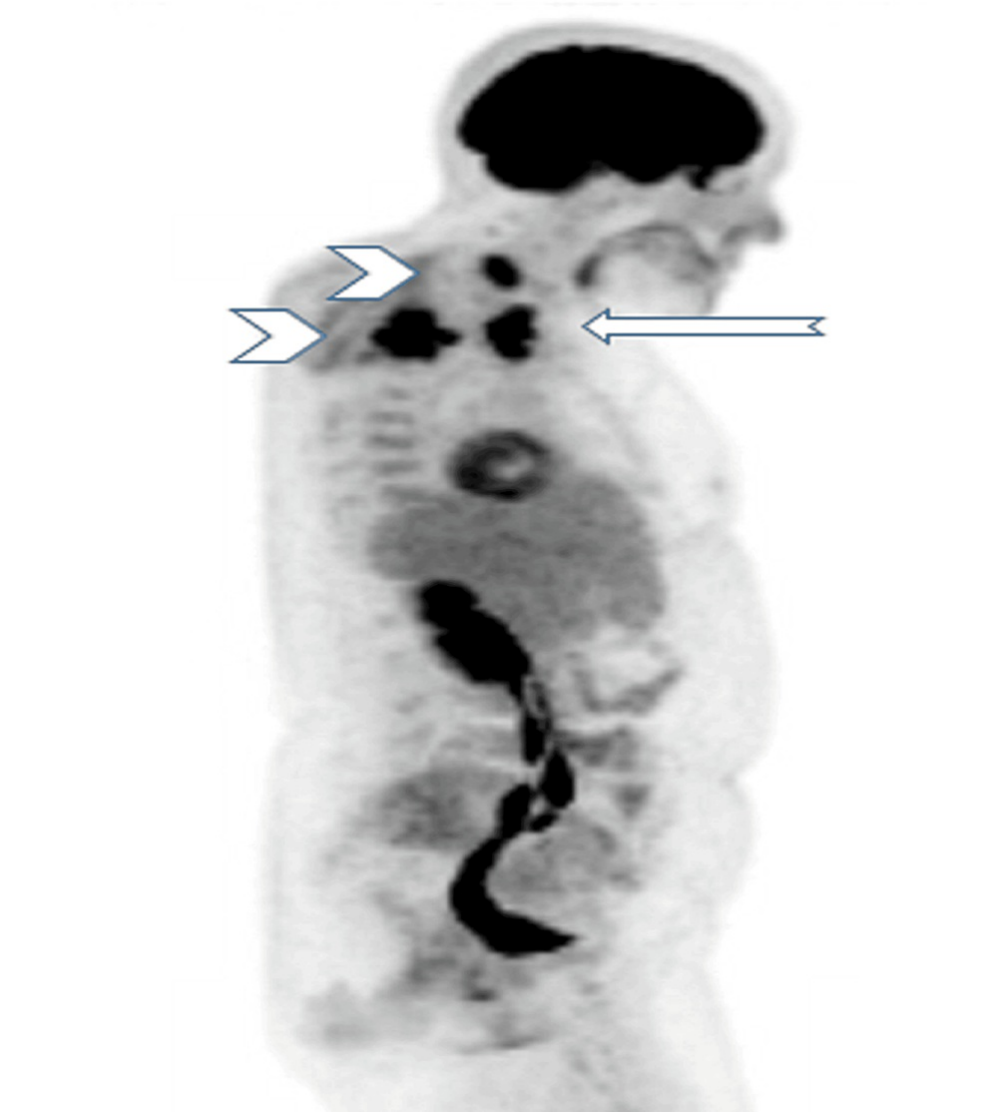
FDG uptake in the upper mediastinal mass (arrow) and two vertebral metastases (arrowheads). FDG: fluorodeoxyglucose

The fluorodeoxyglucose-positron emission tomography (FDG-PET) scan showed hypermetabolic foci in the upper mediastinum with an SUV max of 25 (Figures 1-3) and postoperative changes in posterior decompression with rod and pedicle screw fixation at T1-T4 with metastatic uptake throughout the T2 vertebral body and C6 vertebral body.

A total thyroidectomy was performed. Pathology of the resected thyroid showed foci of benign adenomatoid nodules with 1.5-2 mm oncocytic adenoma in the left lobe. No malignant changes were seen in the resected thyroid. The upper mediastinal mass was not resected due to proximity to vascular and neuronal structures (around the carotid artery and internal jugular vein and the vagus nerve). Fine needle aspiration (FNA) of the upper mediastinal mass was consistent with papillary thyroid cancer. Molecular testing from the FNA sample using Thyroseq V3 showed SQSTM1-NTRK3 chromosomal rearrangement.

**Figure 4:**
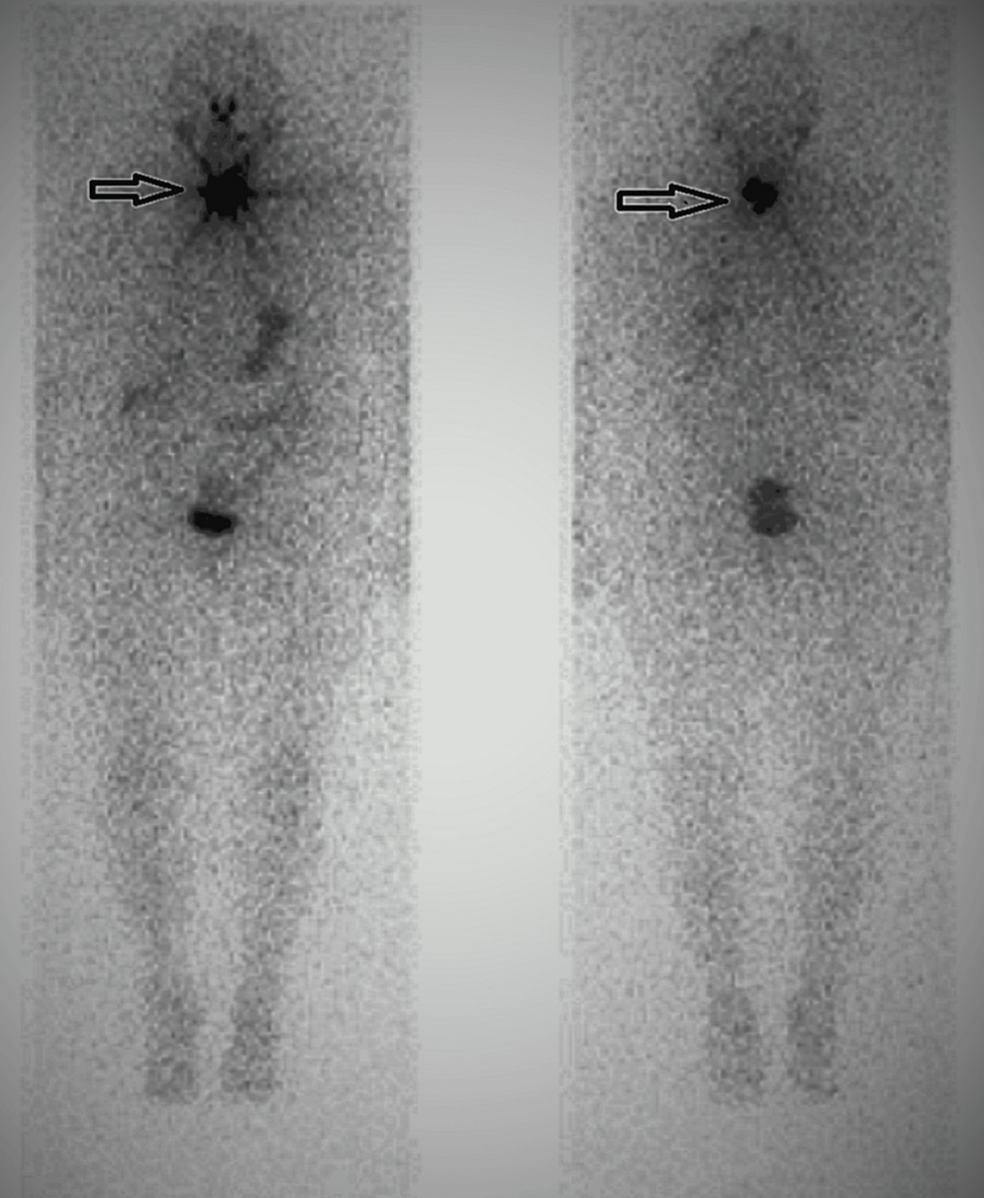
I-123 whole-body scan with uptake in the thyroid bed (arrow).

A recombinant TSH-stimulated I-123 scan status post-thyroidectomy showed intense uptake in the neck area, which most likely represented remnant thyroid tissue. Uptake in the mediastinal mass could not be demonstrated clearly due to proximity to the thyroid bed (Figure 4). Single-photon emission computed tomography (SPECT) was not performed with the radioactive iodine study. The patient underwent removal of the upper mediastinal mass with lymph node dissection at a referral center. Pathology of the mediastinal mass showed a papillary thyroid carcinoma with focal tall cell features, forming a 4 × 2.5 × 2.5 cm mass. The mass demonstrated focal necrosis and extensive invasion into the fibroadipose tissue with invasion of the vagus nerve. The pathologist noted that no definitive background benign thyroid parenchyma was identified, nor was the tumor obviously involving a lymph node. The patient underwent treatment with radioactive iodine I-131 at a dose of 100 millicuries (mci). A posttreatment whole-body scan showed no evidence of metastasis.

The thyroglobulin level decreased from a baseline of 15272 ng/ml to 1857 ng/ml post-surgery and after radioactive iodine ablation and external beam radiation. The patient remained stable with no gross recurrence on follow-up at 14 months post-initial presentation.

## Discussion

Ectopic thyroid tissue is uncommon. It has a prevalence of approximately 1 per 100 000-300 000 people, and it is more prevalent in individuals with thyroid disease, with a rate of approximately 1 per 4000-8000 patients [5]. More than 90% of ectopic thyroid cases are found along the origin and pathway of descent of the thyroid anlage, comprising the area from the base of the tongue to the natural location of the developed thyroid gland. Autopsies performed have shown an increased prevalence of ectopic thyroid tissue estimated in as many as 10% of individuals [5]. Examples of primary ectopic thyroid tissue ranging from the lingual thyroid, thyroglossal duct cyst, mediastinal, lateral aberrant thyroid tissue, and struma ovarii have been found. Ectopic tissue has also been seen in the gallbladder [6] and adrenal glands [7].

Ectopic thyroid tissue is found in the thoracic cavity in approximately 1% of reported ectopic thyroid cases [5]. Ectopic intrathoracic thyroid tissue, in the majority of cases, tends to be benign [4]. The most common signs and symptoms of intrathoracic goiter are dyspnea and coughing due to compression and involvement of the trachea and lung parenchyma. Additional symptoms that have been reported with intrathoracic goiters include dysphagia and superior vena cava syndrome [2-4].

When an intrathoracic mass is first observed, there are a few approaches to care. A commonly used diagnostic technique is FNA, which has been used in varying intrathoracic masses. Maintaining suspicion that these masses could represent ectopic thyroid tissue is important to making the diagnosis. Sending the thyroglobulin level from the biopsy needle washout can facilitate the diagnosis and staining the specimen for thyroid transcription factor 1 (TTF1) or thyroglobulin can be used to direct further care.

Other commonly used diagnostic tests include radioactive iodine uptake scans, preferably with SPECT, to facilitate precise localization.

There appears to be no consensus about the optimal therapeutic strategy, likely due to the limited number of cases. Excision of an intrathoracic mass is a very definitive diagnostic approach, as well as a therapeutic approach, in most cases. For biopsy-proven benign intrathoracic ectopic thyroid tissue with minimal or no symptoms, observation and repeat imaging can be used.

There are very few instances when this ectopic tissue is found to be cancerous. To investigate this matter further, we conducted a literature review of cases of primary intrathoracic ectopic thyroid carcinomas. In the review, we found, including our own, 37 cases reported [4,8-16]. Some of these articles were abstracts only, and others had only limited data available. A summary of these cases is provided in Table 1.

**Table 1:** Characteristics of Reported Cases of Ectopic Intrathoracic Thyroid Carcinomas (n = 36)

Source	Number of participants	Age	Sex	Ectopic intrathoracic carcinoma histological type	Metastasis at presentation
Barrea et al. [8]	1	N/A	N/A	Papillary	No
Hirnle et al. [9]	1	55	F	Follicular	N/A
Ma et al. [10]	1	67	F	Papillary	N/A
Mishriki et al. [11]	1	N/A	N/A	Hürthle cell tumor	Regional spread to the lungs
Nakaya et al. [4]	5	N/A	N/A	Follicular (2), Papillary (3)	N/A
Nervi et al. [12]	23	Mean (63) for 9/23-14 N/A	F (8) M (1) N/A (14)	Anaplastic (3), Follicular (11), Papillary (6), Medullary (3)	N/A
Sand et al. [13]	1	62	F	Papillary	N/A
Shafiee et al. [14]	1	39	F	Papillary	No
Shah et al. [15]	1	45	F	Papillary	N/A
Vázquez et al. [16]	1	68	F	Papillary	Regional spread to the clavicle

There were four studies that had incomplete or partially incomplete data available. The following statistics were calculated based on the available data. Intrathoracic carcinomas occurred at a higher frequency in women, at 88% (14/16). The average age at diagnosis was 61 years old. The ages ranged from 39 to 73 years old. The types of ectopic thyroid carcinomas were papillary (43%, 16/37), follicular (38%, 14/37), anaplastic (8%, 3/37), medullary (8%, 3/37), and Hürthle cell (3%, 1/38). Two of the cases reported regional spread of the carcinoma into surrounding tissue. To the best of our knowledge, our patient appears to be the first reported case of an ectopic primary intrathoracic thyroid carcinoma that had distant metastasis.

We did not find molecular testing performed on pathology or cytology specimens in any of the reported cases or case series. Molecular testing in our patient showed SQSTM1-NTRK3 chromosomal rearrangement. The SQSTM1-NTRK3 chromosomal rearrangement has been described in the literature in thyroid cancer but not in carcinoma originating from ectopic thyroid tissue [17].

In thyroid tissue, the MAPK signaling pathway is activated after the binding of growth factors to tyrosine kinase receptors. This binding triggers intracellular signaling to activate cell growth, differentiation, and other important cellular processes. Differentiated thyroid cancer, such as papillary and follicular thyroid carcinoma, can result from mutations in the MAPK signaling pathway, including mutations in neurotrophic tropomyosin receptor kinase (NTRK), BRAF, RAS, and RET/PTC [1]. The use of molecular diagnostics is becoming a very important factor in improving the outcomes of patients with malignancies. A differentiated thyroid cancer with an SQSTM1-NTRK3 chromosomal rearrangement involves the fusion of the 5’ portion of sequestosome 1 (SQSTM1) to neurotrophic tyrosine receptor kinase 3 (NTRK3). The resulting fusion causes constitutive activation of NTRK3 in the RAS-RAF-MEK-ERK pathway. This type of differentiated thyroid cancer could be targeted by medications such as larotrectinib and endtrectinib [17].

## Conclusions

This case highlights the possibility of developing thyroid cancer in ectopic thyroid tissue and the unique challenges in the diagnosis and management of such a rare disease. Our patient had a very aggressive disease presentation that required multimodal treatment, including thoracic spine decompression, total thyroidectomy, mediastinal ectopic thyroid cancer resection, external beam radiation to the affected spine area, and high-dose radioactive iodine treatment. The use of molecular diagnostics is very important to improving the outcomes in such patients. The tumor had SQSTM1-NTRK3 chromosomal rearrangement. This fusion can be targeted by medications such as larotrectinib and endtrectinib.
